# CRISPR/Cas9-Based Functional Characterization of SfUGT50A15 Reveals Its Roles in the Resistance of *Spodoptera frugiperda* to Chlorantraniliprole, Emamectin Benzoate, and Benzoxazinoids

**DOI:** 10.3390/insects15050314

**Published:** 2024-04-26

**Authors:** Zhan Shi, Mei Luo, Jinxi Yuan, Bin Gao, Minghuan Yang, Guirong Wang

**Affiliations:** 1 Shenzhen Branch, Guangdong Laboratory of Lingnan Modern Agriculture, Genome Analysis Laboratory of the Ministry of Agriculture and Rural Affairs, Agricultural Genomics Institute at Shenzhen, Chinese Academy of Agricultural Sciences, Shenzhen 518120, China; shizhan0522@163.com (Z.S.); meiluo0522@126.com (M.L.); yuanjinxi1022@163.com (J.Y.); gaob1n@163.com (B.G.); yangminghuan1785@163.com (M.Y.); 2 School of Life Sciences, Henan University, Kaifeng 475004, China; 3 Shenzhen Research Institute, Henan University, Shenzhen 518000, China; 4 Guangxi Key Laboratory of Agri-Environmental and Agri-Products Safety, College of Agriculture, Guangxi University, Nanning 530004, China; 5 Key Laboratory of Sustainable Forest Ecosystem Management—Ministry of Education, Northeast Forestry University, Harbin 150040, China

**Keywords:** *Spodoptera frugiperda*, *CRISPR/Cas9*, insecticides, benzoxazinoids

## Abstract

**Simple Summary:**

Uridine diphosphate (UDP)-glycosyltransferases (UGTs) are significant phase II detoxification enzymes in insects. In this study, we have utilized CRISPR/Cas9 to generate *SfUGT50A15* knockout strains to explore its possible function in governing sensitivity to chemical insecticides or benzoxazinoids. The bioassay results indicated that the *SfUGT50A15* knockout strains were significantly more sensitive to chlorantraniliprole, emamectin benzoate, and benzoxazinoids than the wild-type strains. This finding highlights the involvement of *SfUGT50A15* in the resistance of *Spodoptera frugiperda* to chlorantraniliprole, emamectin benzoate, and benzoxazinoids. This study not only presents new molecular targets for controlling *S. frugiperda* but also establishes a foundation for the control of lepidopteran pests.

**Abstract:**

UDP-glycosyltransferases (UGTs) are a diverse superfamily of enzymes. Insects utilize uridine diphosphate-glucose (UDP-glucose) as a glycosyl donor for glycosylation in vivo, involved in the glycosylation of lipophilic endosymbionts and xenobiotics, including phytotoxins. UGTs act as second-stage detoxification metabolizing enzymes, which are essential for the detoxification metabolism of insecticides and benzoxazine compounds. However, the UGT genes responsible for specific glycosylation functions in *S. frugiperda* are unclear at present. In this study, we utilized CRISPR/Cas9 to produce a *SfUGT50A15-KO* strain to explore its possible function in governing sensitivity to chemical insecticides or benzoxazinoids. The bioassay results suggested that the *SfUGT50A15-KO* strain was significantly more sensitive to chlorantraniliprole, emamectin benzoate, and benzoxazinoids than the wild-type strains. This finding suggests that the overexpression of the *SfUGT50A15* gene may be linked to *S. frugiperda* resistance to pesticides (chlorantraniliprole and emamectin benzoate) as well as benzoxazinoids (BXDs).

## 1. Introduction

*Spodoptera frugiperda* (Lepidoptera: Noctuidae) is a notorious invasive pest characterized by its broad host range, high fecundity, and high adaptability [1]. It can infest 353 plant species, including important crops such as maize (*Zea mays*), rice (*Oryza sativus*), cotton (*Gossypium hirsutum*), and sorghum (*Sorghum bicolor*) [2]. *Spodoptera frugiperda* is native to the tropical and subtropical regions of the Americas [3]. Following its invasion of Africa in 2016, it has spread to 44 countries throughout the continent in just two years [4]. Since invading China in January 2019, it has expanded across 26 provinces [5]. Currently, the utilization of chemical pesticides is the primary approach for controlling *S. frugiperda* [6]. However, the widespread use of chemical pesticides has resulted in genetic resistance, lowering the efficiency of common chemical pesticides. Therefore, understanding the genetic mechanism of pesticide resistance in *S. frugiperda* is critical for the efficient chemical management of this pest [7,8].

UDP-glycosyltransferases (UGTs) are a class of multifunctional enzymes, widely found in viruses, bacteria, fungi, plants, and animals [9]. UGTs primarily use UDP-glucuronic acid as a sugar donor in mammals, while UDP-glucose is used as a sugar donor in plants and insects [10]. Many studies have demonstrated that UGTs are located in various insect organs such as the fat body, midgut, and antennae [10,11,12]. Insect UGTs are important components of the phase II detoxification metabolizing enzymes, which have a crucial role in the metabolism of exogenous substances, hlorantraniliprole, tebufenozide, indoxacarb, chlorfenapyr, spinosyns, abamectin, and metaflumizone [13,14,15]. For instance, the expression levels of the genes *UGT40R3* and *UGT46A6* were significantly up-regulated following insecticide treatment in *Spodoptera mauritia* [11]. The suppression of the genes *UGT40D17*, *UGT40F20*, and *UGT40R18* in *S. frugiperda* significantly elevates the insensitivity of field populations to chlorpyrifos and chlorfenapyr [16].

Besides chemical pesticides, it has been reported that UGTs of *S. frugiperda* can detoxify the predominant secondary metabolite of maize, benzoxazinoids (BXDs), which have been reported to suppress the growth of various insect herbivores of maize [17,18,19]. In maize, BXDs are constitutively stored in vacuoles as inactive glucosides. Upon insect attack, the disruption of maize tissues can lead to the exposure of BXD glucosides to their specific β-glucosidases, causing the release of the active aglucones and their toxic degradation products [20,21]. In *S. frugiperda*, *SfUGT33F28* and *SfUGT40L8* can re-glycosylate toxic benzoxazinoids into non-toxic stable glucosides to detoxify benzoxazinoids [22].

The UGT50 family is the most conserved among the UGT families and is considered the ancestor UGT family in insects [10]. However, functional studies on the UGT50 family in insects have not been reported. In this study, a *SfUGT50A15* knockout strain (*SfUGT50A15-KO*) was developed using CRISPR/Cas9 technology to investigate its function in tolerance towards chemical pesticides and benzoxazinoids. In the results, the knockout strain exhibited higher mortality rates than the wild-type in the presence of chlorantraniliprole and emamectin benzoate. This strain also grew slower than the wild-type on maize tissues, but this difference was absent with a mutated maize genotype with minimal BXD. These findings indicate that *SfUGT50A15* contributes to the detoxification of chemical pesticides and plant defense metabolites. This provides new insights into the function of the ancestral UGT gene family UGT50 in insects.

## 2. Materials and Methods

### 2.1. Insects

The experimental *S. frugiperda* colony was collected from Ruili, Yunnan Province, China, in December 2019 and maintained in laboratory environments since then. The *S. frugiperda* larvae were grown on artificial diets [23] under routine laboratory conditions (a temperature of 27 ± 1 °C, humidity of 65 ± 5%, and a photoperiod of 14 h of light and 10 h of darkness). Adults were fed on 10% sucrose solution and were not exposed to any insecticides or benzoxazinoids during rearing.

### 2.2. Insecticides and Plants

The insecticides utilized in the bioassay (chlorantraniliprole, emamectin benzoate, chlorantraniliprole, spinetoram, lufenuron) were purchased from Mannhage (Shanghai, China) Biotechnology Company, and other reagents employed in the experiments were analytically pure. Two maize strains were utilized in this study, the BX knockout mutant BX2 (bx2::Ds) and the wild-type maize strain W22. Plants were grown for two weeks in a growth chamber under a controlled photoperiod of 16 h of light and 8 h of darkness, and 180 mm^−2^/s^−1^ of photons at a constant temperature of 23 °C and 60% humidity. The maize strains utilized in this study were provided by Dr. Shaoqun Zhou’s lab.

### 2.3. Characterization and Cluster Analysis of SfUGT50A15 Sequences

The cDNA sequence of the *SfUGT50A15* gene was queried based on the transcriptome and genome databases of *S. frugiperda* (XP_035437704.2). The UGT50A protein sequences of other Lepidopteran species (*Bombyx mori*, *Zygaena filipendulae*, *Tuta absoluta*, *Plutella xylostella*, *Chilo suppressalis*, *Helicoverpa armigera*, and *Spodoptera exigua*) were retrieved and downloaded from the NCBI database (https://blast.ncbi.nlm.nih.gov/Blast.cgi, accessed on 1 December 2023). Multiple alignments were performed for eight UGT50A genes using MAFFT v7.487 (https://mafft.cbrc.jp/align-ment/soft-ware/, accessed on 1 December 2023) and the phylogenetic tree was constructed using FastTree v2.1.10 with default parameters [24]. The tree was visualized by EvolView (https://www.evolgenius.info/evolview-v2/, accessed on 1 December 2023) ProtParam (https://web.expasy.org/protparam/, accessed on 1 December 2023) was utilized to examine the physicochemical properties of amino acids. SOPMA (NPS@: SOPMA secondary structure prediction (ibcp. fr)) was utilized to predict the secondary structure. TMHMM Server 2.0 (https://services.healthtech.dtu.dk/service.php?TMHMM-2.0, accessed on 1 December 2023) was employed to predict transmembrane structural domains.

### 2.4. Extraction of Total RNA and Synthesis of cDNA

The complete body of *S. frugiperda* individuals across different developmental stages, various tissues of 5th-instar larvae including fat body (FB), midgut (MG), marsupial tube (MT), salivary gland (SA), spermathecae (VD), head (H), and epidermis (EP) were obtained, and total RNA was extracted using the Trizol reagent (Ambion) [25]. The total RNA was reverse transcribed to produce cDNA using HiScript III 1st Strand cDNA Synthesis Kit (Vazyme, Nanjing, China, Cat. No. R312-02), following the manufacturer’s instructions.

### 2.5. Cloning and Analysis of the SfUGT50A15 Gene

To confirm the identity of the full-length sequence of the *SfUGT50A15* gene, specific primers were designed according to the cDNA sequence (Table 1). PCR products were run on a 1.5% agarose gel, and the bands were recovered and purified through the use of an AxyPrep™ DNA Gel Extraction Kit (Axygen, Suzhou, China). The purified PCR products were subcloned into the pEASY-Blunt (pEASY^®^-Blunt Cloning Kit, TransGen, Beijing, China, Cat. No. CB101-01) vector for sequencing. Sequence accuracy was characterized by comparing genomic and cDNA sequences.

### 2.6. Quantitative Real-Time PCR

To characterize the stage and tissue expression profile of *SfUGT50A15*, RNA templates were generated from larval tissues at different developmental stages. The specific primers (Table 1) for qPCR of the *SfUGT50A15* gene were designed using Primer Premier 5.0 software. *SfActin* was utilized as the reference gene to characterize the period and tissue expression of *SfUGT50A15* by qRT-PCR, and analyzed using triplicate experiments. The results were analyzed using the 2^−(∆∆Ct)^ method [26].

### 2.7. Design and Preparation of sgRNA for CRISPR/Cas9 

According to the CRISPR/Cas9 target design principle 5′-20bp-NGG-3′, the ORF region of the *SfUGT50A15* gene was used as a specific target with the online software CRISPOR (http://crispor.tefor.net accessed on 1 September 2023). The sgRNA site sequence (5′-TACTCCGTCACCCCATTCTTTGG-3′) was constructed and screened on the fifth exon. The selected sgRNA was compared to the *S. frugiperda* genome, and no potential off-target site was identified. A pair of primers (Table 1) was designed that flanked the editing site for edited allele screening at the genomic level. The sgRNA was synthesized according to the manufacturer’s instructions and transcribed in vitro using the GeneArt Precision gRNA Synthesis Kit (Thermo Fisher Scientific, Waltham, MA, USA, Cat. No. A29377). The Cas9 protein (TrueCutTM Cas9 Protein v2) was acquired from Thermo Fisher Scientific.

### 2.8. Egg Collection and Microinjection

Fresh eggs within 1 h of laying were rinsed and treated with sterile water and attached to slides using double-sided adhesive tape. Samples of 8 μL of 300 ng/μL *SfUGT50A15* sgRNA, 1 μL of 5mg/mL Cas9 protein, and 1 μL of phenol red solution were combined (a total 10 μL system), and 2 nL of the mixture was injected into each egg via microinjection (Nanoject III, Drummond, NC, USA). The microinjected eggs (G0 generation) were maintained at a temperature of 27 ± 1 °C and a humidity of 65 ± 5% until hatching.

### 2.9. Phenotypic Observation and Mutation Analysis

The G0 generation of *S. frugiperda* was counted for hatching and mutation rates. One of the midfeet was removed at the adult stage, and genomic DNA was extracted using the PURELINK Genomic DNA Kit (Invitrogen, Waltham, MA, USA, Cat. No. K182001) following the product instructions, and gene fragments containing sgRNA targets were amplified with gene-specific primers (Table 1). The amplification conditions were established as one cycle of 98 °C for 3 min, followed by 35 cycles of 98 °C for 10 s, 56 °C for 15 s, and 72 °C for 15 s, and ultimately a final extension period at 72 °C for 10 min. The quality of amplification products was examined using agarose gel electrophoresis. The PCR products were sequenced, and mutant individuals were identified according to the double sequencing peaks. Following the screening of heterozygous mutants in the G1 generation, individuals of the G2 generation were obtained through mating. Subsequently, homozygous mutants of the G2 generation underwent screening. These homozygous mutants from the G2 generation serve as the basis for population expansion. Individuals homozygous for mutants in the G4 generation and beyond are designated for bioassay experiments.

### 2.10. Fitness Cost Analysis

Egg masses laid on the same day by the wild-type strain and the *SfUGT50A15-KO* strain were harvested and maintained in the same environment to hatch. Each strain was selected as a group of 30 first-hatched larvae, repeated in three groups. Each first-hatched larva was caged individually in a small box and reared on artificial feed until pupation. After fledging, five pairs of adults were randomly chosen from each strain for pairing. The number of eggs laid and the number of eggs hatched were counted. During the experiment, the larval stage, pupal weight, eclosion time, lifespan of adult, egg production and hatching rate were observed and counted.

### 2.11. Insect Bioassay

The toxicity of five pesticides towards wild-type *S. frugiperda* and the *SfUGT50A15* knockout strain were characterized by dosing the artificial diet [27]. The pesticide-dosed diet was sectioned into approximately 5 mm^3^ cubes and placed in 24-well plates, with one third-instar larva kept in each well. Mortality was identified after two days, and the larvae were considered dead if they did not respond to gentle touching with a brush, or if there were obvious symptoms of poisoning (e.g., deformities, convulsions). Mortality data were used to compute the insecticide concentration killing 50% of the larvae and the corresponding 95% confidence limits. LC_50_ values were considered significantly different if the confidence limits did not overlap [28].

Toxicological treatments were performed using the feed mixing method, where the pesticides were sequentially diluted in sterile water to four concentrations (1 μg/g, 2 μg/g, 4 μg/g, 8 μg/g), and mortality was documented after 48 h. Four sets of biological replicates for each concentration were employed, with each replicate containing 48 3rd-instar larvae that hatched on the same day.

Both W22 and *bx2::Ds* maize seedlings were planted. Fourteen days following seedling emergence, neonates of wild-type *S. frugiperda* and the *SfUGT50A15* knockout strain were gently placed on seedling leaves (three neonates per seedling), and caged with a plastic box. Each larva was weighed after seven days of feeding.

### 2.12. Statistical Analysis

Biological data were analyzed using IBM SPSS Statistics 23.0 software (IBM Corp., Armonk, NY, USA). Data are presented as mean ± standard error. Two treatments were compared using Student’s *t*-test with normal distributions. Statistical significance was determined using one-way ANOVA with LSD post-test (*p* ≤ 0.05 was considered significant) in different treatments [29]. Figures were prepared using GraphPad Prism 8.0 (https://www.graphpad.com, accessed on 1 December 2023).

## 3. Results

### 3.1. Sequence and Phylogenetic Analysis of the UGT Gene

**Figure 1:**
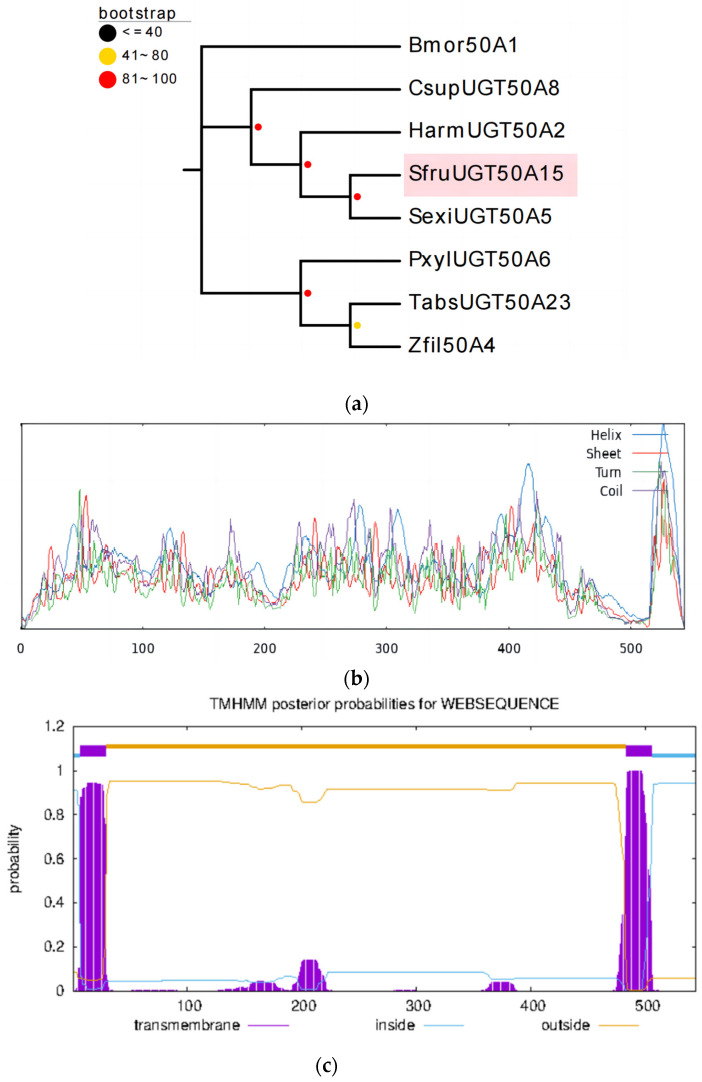
Bioinformatics analysis of SfUGT50A15. (**a**) Phylogenetic analysis of *SfUGT50A15* protein and its homologs. Bmor-*Bombyx mori*, Csup-*Chilo suppressalis*, Harm-*Helicoverpa armigera*, Sfru-*S. frugiperda*, Sexi-*Spodoptera exigua*, Pxyl-*Plutella xylostella*, Tabs-*Tuta absoluta*, Zfil-*Zygaena filipendulae*. (**b**) *SfUGT50A15* secondary structure prediction peaks. Blue: alpha helix, green: beta turn, yellow: random coil, red: extended strand. (**c**) Predicted transmembrane domains of *SfUGT50A15* protein.

The *SfUGT50A15* gene sequence is 1635 bp in length and encodes 544 amino acids. In the generated phylogenetic tree of UGT, *S. frugiperda* was more homologous with *S. exigua*, with a high homology and close affinity, followed by a somewhat close affinity with *H. armigera*, supported by a good self-expansion value (100%), indicating a short differentiation time and lower variation (Figure 1a). The predicted secondary structure of *SfUGT50A15* showed that the protein contained 35.29% random coils, 47.98% alpha helixes, 3.68% beta-turns, and 13.05% extended strands (Figure 1b). This protein is predicted to contain two transmembrane domains (Figure 1c).

### 3.2. Expression Profiles of SfUGT50A15

**Figure 2:**
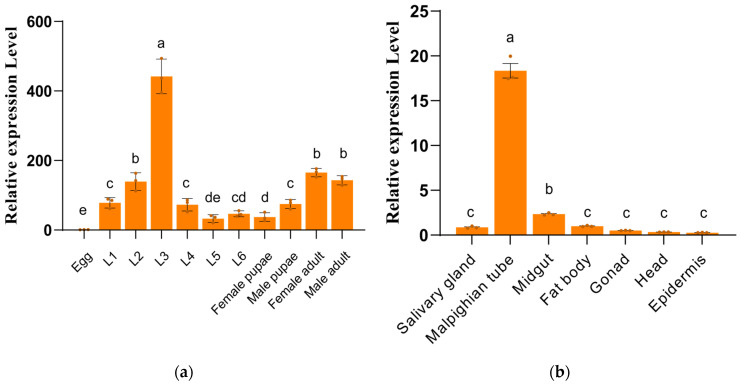
Relative expression levels of the *SfUGT50A15* gene in *S. frugiperda*. (**a**) Different developmental stages. L1-L6, 1st-6th-instar larvae. (**b**) Different tissues; Mean ± SE from three replicates is shown. Different letters above bars indicate significant differences (*p* < 0.05) according to the Student’s *t*-test.

We employed quantitative real-time PCR to examine the expression pattern of *SfUGT50A15* across various developmental stages and tissue types. The mRNA levels of *SfUGT50A15* increased gradually from the egg stage to the third-instar larvae, were reduced from the third to the sixth instar, and significantly increased following the pupal stage in adult females (Figure 2a). In the tissue expression profile of the larvae, *SfUGT50A15* expression was highest in the Malpighian tube (Figure 2b).

### 3.3. Generation of the SfUGT50A15-KO Line

**Figure 3:**
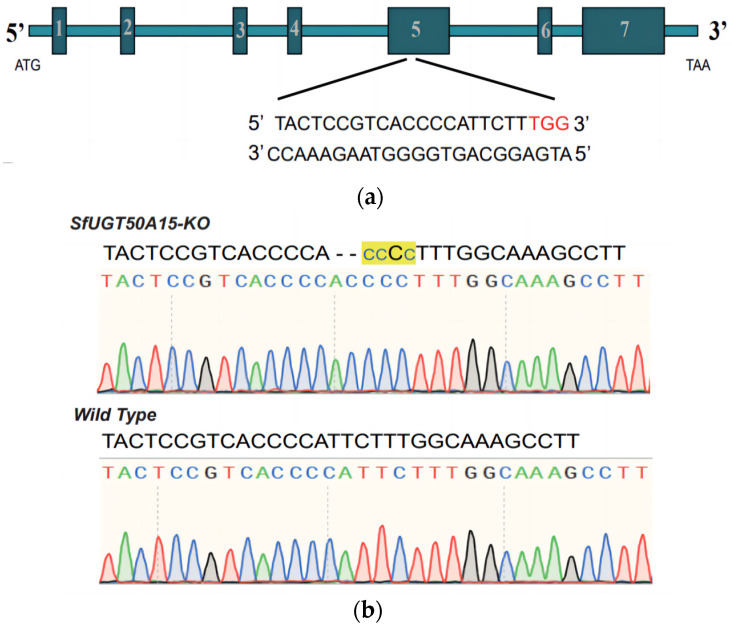
Schematic representation of the *SfUGT50A15* knockout. (**a**) Target location of sgRNA. The *SfUGT50A15* gene has seven exons and the sgRNA was designed to be in exon 5; the target sequence is shown below the fold. (**b**) Representative sequencing chromatograms of PCR products from wild-type (WT) and G0 injected eggs. Sequences determined by TA clone sequencing are shown at the top of the image. In the mutant sequence *SfUGT50A15-KO*, the deletion is shown as a dash, the insertion as a blue lowercase letter, and the yellow shading as the mutated region, totaling 2 bp for the deletion and 3 bp for the insertion.

A strain with a 3 bp deletion and a 2 bp insertion was selected from the mutations detected (Figure 3). The G1 generation larvae obtained by crossing G0-generation moths with the wild-type strain were reared to adulthood, and the midfeet of the adults were harvested for sequencing. Three hundred G1-generation adults of the same mutation type (3 bp deletion and a 2 bp insertion) were obtained. These adults were harvested and crossed with wild-type individuals to obtain the G2 generation. Three hundred individuals from the G2 group were genotyped using target genomic sequencing, of which 45.3% (136/300) were homozygous. These homozygous adult individuals were maintained in the same cage and mated to generate the *SfUGT50A15* knockout strain (*SfUGT50A15-KO*).

### 3.4. No Fitness Costs Were Associated with the Knockout

**Figure 4:**
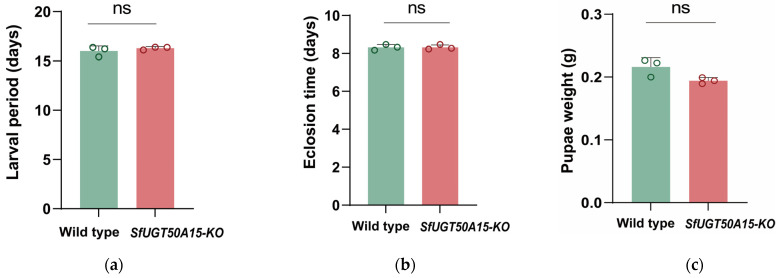

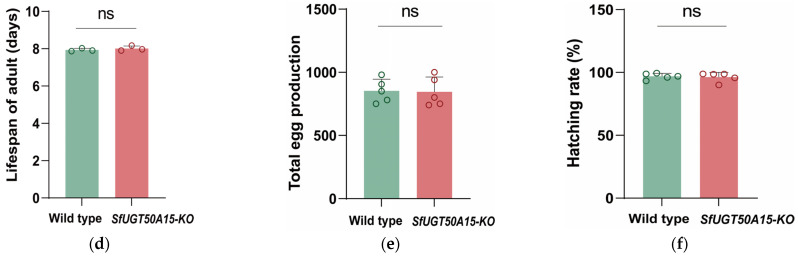
Statistics on biological characteristics of *S. frugiperda* one generation after *SfUGT50A15* gene knockout. (**a**) Larval period. (**b**) Eclosion time. (**c**) Pupal weight. (**d**) Lifespan of adult. (**e**) Total egg production. (**f**) Hatching rate. ns, not significant.

We examined the cost of the adaptation of the *SfUGT50A15* knockout strain. As a result, there were no significant differences in the larval period, eclosion time, pupae weight, adult lifespan, fecundity, or egg hatching rate between the two genotypes (Figure 4).

### 3.5. Effect of SfUGT50A15 Knockout on the Susceptibility of S. frugiperda Larvae to Insecticides

The susceptibility of third-instar larvae to chlorantraniliprole and emamectin benzoate in the *SfUGT50A15-KO* line was significantly elevated (according to non-overlapping confidence limits) by 5.67 and 5.00 times, respectively (based on LC_50_ values). The results of the bioassays using other insecticides exhibited no significant change in the susceptibility of the *SfUGT50A15-KO* larvae compared to the wild-type larvae (Table 2).

**Figure 5:**
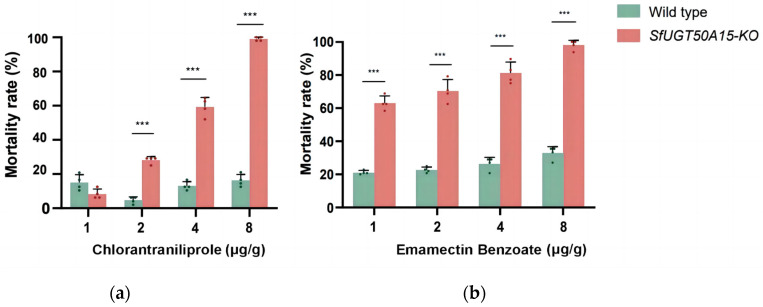
Mortality of wild-type *S. frugiperda* and *SfUGT50A15* knockout strain at different concentrations of (**a**) chlorantraniliprole and (**b**) emamectin benzoate. ***, *p* < 0.001.

The bioassay findings indicated that the greatest difference in the mortality between the wild-type strain and *SfUGT50A15-KO* strain was found after treatment with chloramectin benzoate, followed by emamectin benzoate. For chlorantraniliprole, the mortality rate of *SfUGT50A15-KO* was consistently higher than that of the wild-type (Figure 5a). For emamectin benzoate, *SfUGT50A15-KO* had a similar mortality rate as that of the wild-type at 1 µg/g. However, at higher concentrations, the mortality rate of the *SfUGT50A15-KO* larvae was significantly higher than that of the wild-type (Figure 5b).

### 3.6. Susceptibility of Larvae to Benzoxazinoids Following SfUGT50A15 Knockout

**Figure 6:**
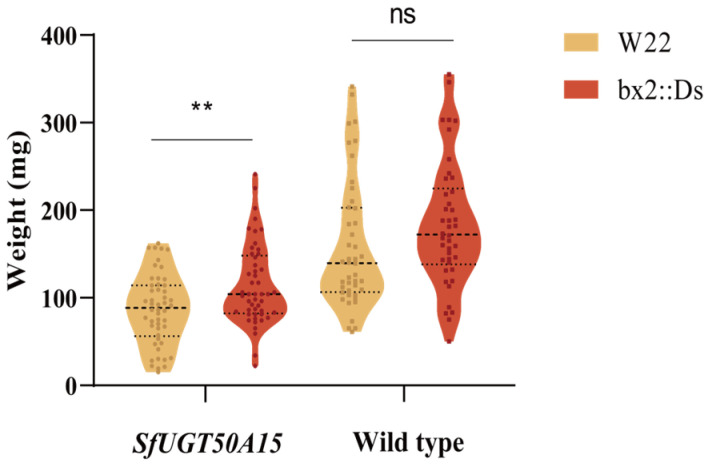
Body weight of *S. frugiperda* larvae after feeding for 7 days on W22 and BX2 strains of maize. **, *p* < 0.01. ns, not significant.

To characterize the role of *SfUGT50A15* in the detoxification of benzoxazinoids, we performed bioassays using a BXD-deficient mutant line, *bx2::Ds*, and its wild-type progenitor, W22. Whereas the wild-type larvae grew similarly on the W22 and the *bx2::Ds* plants, the *SfUGT50A15-KO* larvae grew significantly better on the *bx2::Ds* plants than the W22 plants (Figure 6). This indicates that the *SfUGT50A15* gene is important in the tolerance of *S. frugiperda* to benzoxazinoids.

## 4. Discussion

Glucuronidation is a major reaction catalyzed by the UGT family, which is associated with the detoxification process and that is reported in many insect genomes, like *Bemisia tabaci* [18], *P. xylostella* [19], *S. exigua* [30], *S. litura* [31], and *S. frugiperda* [32]. The function of UGTs in drug resistance in insects has attracted increasing attention, as UGT genes are associated with insects’ detoxification of exogenous substances. However, functional studies on the tolerance of the UGT50 family are limited. The UGT50 family is the most conserved UGT family in insects, and UGT50A from Lepidoptera is a single-copy gene [10]. Therefore, whether the UGT50A in Lepidoptera could evolve to develop the ability to detoxify plant secondary metabolites and pesticides is the big question in this study.

Previous studies have indicated that UGTs enhance catalytic activity leading to the resistance of *Musca domestica* to insecticides [33]. Through bioassays and transcriptome sequencing, it has been observed that certain UGT mRNAs have shown an upregulation trend, suggesting the potential involvement of UGT in resistance to other classes of insecticides, including pyrethroids, carbamates, and neonicotinoids [34,35]. In Lepidoptera, the role of UGTs has been demonstrated to correlate with detoxification tolerance to insecticides. For instance, chlorantraniliprole significantly induced the expression of *UGT2B17* in susceptible *P. xylostella*. RNA interference with this UGT increased the toxicity of chlorantraniliprole to *P. xylostella* larvae [36]. The inhibitor of UGTs, 5-nitrouracil, heightened the toxicity of indoxacarb against *S. litura*. Additionally, the knockdown of *UGT33J17* and *UGT41D10* reduced the viability of Spli-221 cells and increased the *S. litura* larval susceptibility to indoxacarb [31]. In *Myzus persicae*, the overexpression of *UGT344P2* significantly enhanced field populations’ resistance to sulfoxaflor [34]. Similarly, the overexpression of UGT genes (*UGT386H2*, *UGT386J2*, *UGT386N2*, and *UGT386P1*) in *N. lugens* was associated with resistance to both chlorpyrifos and clothianidin [37].

Many studies have shown that plant secondary metabolites are partially metabolized by UGTs via glycosylation for host adaptation. For example, *UGT41B3* and *UGT40D1* from *H. armigera* facilitate the glycosylation of gossypol, primarily resulting in the formation of the diglycosylated gossypol isomer [38]. Additionally, the depletion of four UGTs, namely, *UGT330A3*, *UGT344D5*, *UGT348A3*, and *UGT349A3*, resulted in a notable increase in the susceptibility of *M. persicae* to nicotine [39]. Furthermore, the UGT33 and UGT40 families in *S. frugiperda*, particularly *UGT33F28* and *UGT40L8*, have demonstrated resistance to the benzoxazines of maize, and the homologous gene *UGT33F28* exhibits conservative functionality in the closely related species *S. littoralis* [22].

In our study, we observed similar findings, in which the knockout of *SfUGT50A15* elevated the toxicity of chlorantraniliprole and emetamectin benzoate towards *S. frugiperda* larvae. We speculate that *S. frugiperda* larvae without the *SfUGT50A15* gene are unable to efficiently excrete chlorantraniliprole, emetamectin benzoate, and benzoxazinoids, resulting in the accumulation of exogenous substances in their bodies and enhancing the susceptibility of *SfUGT50A15* knockout strains. Hormesis is a toxicological phenomenon whereby exposures to low doses of stress results in biological stimulation [40]. In a bioassay, we observed that under low concentrations of chlorantraniliprole treatment, the mortality rate of the wild-type strains was higher than that of the knockout strains. This may be attributed to the induction of a hormetic dose response in the knockout strain of *S. frugiperda* by low concentrations of chlorantraniliprole. The specific mechanisms underlying this phenomenon warrant further investigation.

The CRISPR/Cas9 system has been successfully utilized globally across various fields, enabling the manipulation of multiple target genes in diverse non-model species, such as *S. frugiperda* [41,42]. In this study, the editing frequency of the target site reached 28.2% in the G0 generation. The efficiency of gene editing can be measured by the mutation frequency, but it may be impacted by various factors, including the target gene, the concentration of sgRNA or Cas9, the injection location, and the depth of injection [43,44].

*SfUGT50A15* was highly expressed in the larval Malpighian tube, while its ortholog in *S. exigua* displayed a different tissue-specific expression pattern. The Malpighian tube is the primary organ of excretion in insects. The high expression of *SfUGT50A15* in the Malpighian tube may indicate its involvement in the metabolism and detoxification of xenobiotic substances (insecticides) [45]. Furthermore, the *SfUGT50A15* gene may also be associated with morphogenetic processes linked to the Malpighian tube, and further investigation into biochemical and physiological changes following knockdown is warranted.

A statistical analysis was conducted on the biological characteristics of homozygous knockout strains of *S. frugiperda SfUGT50A15*. The results revealed no significant differences between the *SfUGT50A15* knockout homozygous strains and the wild-type strain in terms of larval duration, eclosion time, pupal weight, adult lifespan, adult oviposition quantity, and egg hatching rate. These findings suggest that *SfUGT50A15* may not be involved in the growth and development of *S. frugiperda*, or its deletion may have led to the compensatory upregulation of other growth-regulating genes [46,47]. Further research is needed to elucidate the specific regulatory mechanisms.

## 5. Conclusions

The results of this study offer new functional evidence that *SfUGT50A15* can participate in the metabolism and detoxification of exogenous substances like chlorantraniliprole, emamectin benzoate (insecticides), and benzoxazinoids, offering a new molecular target for the greening and control of global pests.

## Figures and Tables

**Table 1:** Primer used in this study.

Primer Name	Primer Sequence (5′-3′)	Primer Purpose
SfUGT50A15.qF	ACAGAGCCCTGAATGCCATC	RT-qPCR
SfUGT50A15.qR	TGAAGCTCACATTCTTCGCC	
SfruActinF	TACTCCTAAGCCTGTTGATG	RT-qPCR
SfruActinR	TTATGTCATGGTGCCGAAT	
SfUGT50A15.F1	ACGTAGTAGGGGCAGCCC	Cloning the *SfUGT50A15* gene
SfUGT50A15.R1	TATGTACATTACATTTTATAAACTGAACATCGATC	
SfUGT50A15.sgF	GAAATTAATACGACTCACTATAGGACTCCGTCACCCCATTCTT	Preparation of sgRNA templates
SfUGT50A15.sgR	TTCTAGCTCTAAAACAAGAATGGGGTGACGGAGT	
SfUGT50A15.F2	TCAGGAAGGACTTTTGATCTCG	Identification of somatic mutations
SfUGT50A15.R2	CATTCTGGAGTATGAAGCTCACAT	

**Table 2:** Sensitivity of 3rd-instar larvae of *SfUGT50A15* knockout strain of *S. frugiperda* and wild-type strain to five pesticides.

Insecticide	Strain	LC_50_ (µg/g) ^a^	95% FL ^b^	N ^c^	Toxicity Ratio ^d^
Chlorantraniliprole	WT	19.305	11.08–33.636	192	5.67 ^e^
	*SfUGT50A15-KO*	3.402	1.919–6.032	192	
Emamectin benzoate	WT	15.988	7.803–32.757	192	5.00 ^e^
	*SfUGT50A15-KO*	3.196	1.046–6.228	192	
Spinetoram	WT	0.244	0.244–8.815	192	3.87
	*SfUGT50A15-KO*	0.063	0.063–8.601	192	
Lufenuron	WT	0.137	0.137–5.097	192	1.28
	*SfUGT50A15-KO*	0.107	0.107–3.737	192	
Chlorfenapyr	WT	4.081	4.081–31.146	192	1.12
	*SfUGT50A15-KO*	3.642	3.642–27.171	192	

^a^ Lethal concentration that kills 50% of *S. frugiperda*. ^b^ The 95% fiducial limits of LC_50._^c^ Number of larvae used in the bioassay. ^d^ Toxicity Ratio = LC_50_ value for wild-type strain (WT) divided by LC_50_ value for knockout strain (*SfUGT50A15-KO*). ^e^ The LC_50_ of the wild-type strain of the same pesticide was significantly higher than that of *SfUGT50A15-KO*.

## Data Availability

The original contributions presented in the study are included in the article, further inquiries can be directed to the corresponding author.
